# Challenges in catalyzing and sustaining research in translational science

**DOI:** 10.1017/cts.2023.651

**Published:** 2023-10-13

**Authors:** Mimi Kim, Paul Marantz, Sylvia Suadicani, Sofiya Milman, Marla J. Keller

**Affiliations:** 1 Harold and Muriel Block Institute for Clinical and Translational Research, Albert Einstein College of Medicine, Bronx, NY, USA; 2 Department of Epidemiology and Population Health, Albert Einstein College of Medicine, Bronx, NY, USA; 3 Departments of Urology, Albert Einstein College of Medicine, Bronx, NY, USA; 4 Department of Medicine, Albert Einstein College of Medicine, Bronx, NY, USA

**Keywords:** Translational science, translational research, education, training, pilot projects

## Abstract

Advancing the new field of translational science and developing innovative solutions to overcome translational roadblocks are key priorities of the Clinical and Translational Science Awards (CTSA) Program of the National Center for Advancing Translational Science (NCATS). However, interpreting this emerging concept of “translational science” (TS) as a field of inquiry distinct from “translational research” (TR) and developing real-world investigations in TS can be challenging. The goal of this paper is to share the obstacles the Einstein-Montefiore CTSA hub has faced in generating institutional interest and research in TS and to present potential strategies for addressing them. The aim is to stimulate dialog within the wider CTSA community and beyond about the need to systematically examine how TS should be efficiently and effectively pursued, that is, the science of translational science. The collective sharing of experiences and innovative approaches to overcoming TS challenges that arise at CTSA hubs is critical if the field is to grow and gain wider recognition and acceptance by the scientific and broader communities.

## Introduction

In contrast to translational research (TR), which focuses on advancing knowledge about a specific target or disease, the mission of translational science (TS) is to scrutinize and explain the scientific and operational principles underlying each step of the translational process and address the common causes of research inefficiencies and failures across a range of diseases and conditions [1]. Advancing this new field of TS and developing innovative solutions to overcome translational roadblocks are key priorities of the Clinical and Translational Science Awards (CTSA) Program of the National Center for Advancing Translational Science (NCATS) and will lead to improvements in healthcare and health outcomes by NCATS’ goal of bringing more treatments to all people more quickly [2]. However, interpreting this emerging concept of “translational science” as a field of inquiry distinct from “translational research” and developing real-world investigations in TS have created confusion within and challenges for the CTSA community.

In 2022, leaders of the Institute for Clinical and Translational Research (ICTR) at Albert Einstein College of Medicine and Montefiore Health System submitted an application in response to the new NCATS CTSA Funding Opportunity Announcement (CTSA FOA) [3] to obtain continued CTSA support for the ICTR that was established in 2006. The new CTSA FOA differs from the prior FOAs with respect to both program goals and application structure. The new FOA focuses on advances in TS, and required elements in the application include a Clinical and Translational Science (CTS) Research Program to support TS research projects (“Element E”) and a CTS Pilot Module to support TS pilot projects, among other components.

During the preparation and initiation of our new 7-year CTSA UM1 grant that was awarded in March 2023, we encountered several challenges in our efforts to broadly stimulate TS at our hub. The goal of this paper is to share the obstacles we have faced and present potential strategies for handling them. By doing so, we aim to stimulate dialog within the wider CTSA community and beyond about the need to systematically examine how TS should be efficiently and effectively pursued, that is, to begin to consider the “science of translational science.” This is especially important as an increasing number of CTSA hubs refocus their strategic goals and efforts around TS to align with the new CTSA program priorities.

## Difficulty Translating “Translational Science”

The biggest hurdle we have encountered thus far in motivating investigators to engage in TS has been a lack of awareness and understanding of this emerging discipline. There is persistent and ongoing confusion over the concepts of translation, translational research, and translational science, with the latter two frequently used interchangeably. Over the past 2 years, we have released three internal requests for TS project proposals (TS-RFP), and each required substantial effort by ICTR leaders to educate researchers about the definition and purpose of TS. Our first TS-RFP was announced in August 2021, to solicit project proposals for the *CTS Research Program (Element E)* in our CTSA application. We believed that a crowdsourcing approach for selecting an Element E project was the best way to generate innovative TS ideas that capitalize on existing institutional research strengths. We also viewed it as an opportunity for our leadership team to refine our own respective understanding of the new and poorly understood distinction between TR and TS and to begin to broadly expose investigators to the new TS field. Our TS-RFP explicitly specified that proposals must address truly significant roadblocks in clinical and translational research and yield innovations or insights that are generalizable across diseases to increase the overall efficiency or effectiveness of the translational process. We additionally included the link to the seminal paper on TS by Austin [1] and listed the following examples of TS innovations and focus areas: approaches to reduce barriers to clinical trial recruitment and diversity; novel clinical trial designs; methods to improve data interoperability and transparency; studies to determine how best to build trust between researchers and the community; ways to increase stakeholder engagement effort; and identification of well-curated and appropriately phenotyped biospecimens for translational research.

We received 14 proposals in response to our first TS-RFP for the Element E project, but the majority were too narrowly focused on a specific disease area, that is, TR rather than TS projects. Several had the potential to be TS after substantial feedback from ICTR leadership and rewrites to clarify the TR roadblock of interest and generalizability of future results across diseases, but most proposals were still deemed to be too “risky” because of concerns that CTSA grant reviewers would not view them as TS. The project ultimately chosen for our CTSA application is on studying and reducing researcher-level barriers to enrolling people with disabilities in research. It was selected because it addresses the significant translational barrier of lack of study population diversity, will develop innovations with generalizable applicability to many disease areas, advances the CTSA goal to “*deliver the benefits of translational science to all*,” promotes the NIH goal to increase diversity, equity, inclusion, and accessibility for disability, involves collaborations and partnership with nine other CTSA hubs, and leverages our institution’s existing research programs of excellence in disability. ICTR leadership worked very closely with the study team to develop this project and ensure that it would advance the overall TS goals of the Element E program.

In October 2022, we issued a second call for TS proposals for the *RC2 High Impact Specialized Innovation Programs in Clinical and Translational Science*. This NCATS RC2 program supports the development of unique activities, resources, capabilities, and/or expertise at awarded CTSA UM1 hubs that can have a significant impact on TS [4,5]. Each hub can submit only two RC2 applications per funding cycle and can have no more than two grants awarded at a given time. Our TS-RFP for the RC2 program again included concrete examples of suitable TS topics that were directly from the NCATS RC2 funding announcement: innovations in telehealth, regulatory science, clinical informatics, genetics and genomics, pragmatic trials, dissemination and implementation, rural health and health disparities, community outreach and engagement, and other areas that will significantly improve and accelerate biomedical research across a range of diseases and health conditions. To help further ensure that proposals would be TS-focused, applicants were also asked to provide answers to the following questions:

- What is the significance of the problem/roadblock or gap in TR being addressed and the relevance to the stated goals of the CTSA program [5]?
- How will the proposed program uniquely empower research, generate new hypotheses, or contribute a significant resource, platform, tool, data, or technology that is currently lacking and could help accelerate the development of new therapeutics, devices, and/or diagnostics to improve human health?

ICTR leaders fielded inquiries about the RC2 program from 20 individuals and additionally reached out to specific investigators who were already working in TS-related areas, for example, biostatistics and informatics, to encourage them to submit proposals. In the end, 12 proposals were received for consideration, but many again were disease-specific rather than disease-agnostic. The CTSA UM1 PIs contacted the NCATS RC2 program director to discuss the topics of the four projects considered to be the strongest and to confirm that they fit within the scope of the RC2 program. Two proposals were then selected for development into full RC2 applications. Substantial input from ICTR leadership was again needed, so the novelty of the approaches and clinical and translational science gap areas being addressed were clearly articulated and highlighted in the applications.

Our third internal TS-RFP was announced in January 2023, for pilot projects that would be supported by our new CTSA-funded *Clinical and Translational Science Pilot Module*. Given that many proposals submitted to our prior TS-RFPs were found to be nonresponsive to the TS mission, we implemented additional TS education strategies. We gave presentations about TS to faculty in the larger research-focused departments, created a website with PowerPoint slides about TS [6], and strongly encouraged pre-submission consultations with the pilot program leaders (S. Suadicani and S. Milman) to confirm that projects met program criteria. The TS-RFP emphasized that TR projects focused on crossing a particular step of the translational process specific to *a particular target or disease would not qualify for funding*.

Program leaders held pre-submission consultations with 22 individuals from a range of clinical and basic science departments; 18 pilot project proposals were eventually received. Half were still deemed to be nonresponsive, but the nonresponsiveness rate was much lower among proposals that received pre-submission input from program leaders (25%) compared to those that did not (70%). Applications from clinical departments were primarily from junior faculty with MD/DO degrees (six from assistant professors, two from associate professors, and one from a professor); those from basic science departments were mostly from senior faculty with a PhD degree (two from instructor/assistant professors, two from associate professors, and five from professors). While we were aiming to fund four pilot projects in this initial year, only three were considered sufficiently meritorious and innovative to be approved for funding.

Interestingly, the review process revealed differing opinions even among the review committee members about whether certain projects qualified as TS. They debated whether a study that is organ-specific (e.g., lung and brain), but disease-agnostic within the organ, would be considered TS. For example, an innovative and cost-effective method of noninvasively obtaining samples through “cough capture” could provide important diagnostic, therapeutic, or prognostic information and potentially accelerate the identification of new treatments; however, the focus would be entirely on the lung. Would this work be viewed as a TS innovation? The lack of agreement about this among the pilot project reviewers and CTSA leaders underscored the ongoing confusion about the definition and scope of TS.

## Limited TS Funding Opportunities

Currently, NCATS serves as the primary TS funding source through the CTSA-Element E program, CTSA Pilot Module, and RC2 Specialized Innovation Program. The Element E program can support only a limited number of TS projects at each hub throughout the 7-year lifespan of CTSA UM1 awards; the Pilot Module allocates limited funds for projects annually, while the RC2 program restricts institutions from having more than two concurrently funded grants. The few funding streams for TS pose obstacles to its long-term growth and sustainability as an independent field and will deter junior investigators from engaging in TS, since success in building a career in this field will greatly depend on extramural funding.

Moreover, our TS pilot program supports the generation of preliminary data, refinement of research strategies, demonstration of study feasibility, and establishment of proof-of-concept to support subsequent extramural grant applications. But since there are currently no TS-specific R01 grant mechanisms, it is unclear where those future extramural grant applications should be submitted. Should a TS pilot project be reconfigured in the next phase as a disease-focused TR study to target R01 funding from one of the disease-/body-system-focused NIH institutes and centers? If so, will this grantsmanship maneuvering result in real TS advances? Or should the subsequent stage of a TS pilot study be to demonstrate the utility of new TS technologies in a specific disease area (i.e., a TR study that utilizes TS pilot discoveries) rather than to further develop and refine the TS innovation on a larger scale, given that funding for the latter may not exist through existing grant mechanisms at most NIH institutes or centers?

## Motivating TR Investigators to Pursue TS

Investigators specializing in inherently methodological fields such as biostatistics and informatics or those engaged in later stages of the translational research spectrum, such as implementation science and dissemination, may find it more natural to engage in research in TS. These individuals often collaborate across various disease areas, and their innovations can be more readily positioned as having broader applicability across diseases. However, scientists focused on earlier stages of bench-to-bedside translation, or who have dedicated their careers to advancing knowledge within a specific disease area, may face greater challenges in transitioning their research efforts to TS. Their motivation to embrace TS would again largely depend on the availability of ample TS funding opportunities.

Junior investigators face different challenges as they are training for and establishing their research programs and career trajectories that are often supported through CTSA-funded education, training, and career development programs. NCATS has charged those programs as well with pivoting their training to emphasize TS. Indeed, in response to this new programmatic priority, some have even advocated that new educational competencies in TS should be developed [7]. Several of us and others have objected to this on the basis that established TR competencies already include the skillset required to conduct TS studies, and such a distinction between TR and TS at the level of observable skills is therefore unnecessary, hypergranular, and potentially harmful to the TS field [8].

The more salient challenge faced by our trainees and scholars is how to fashion a research career in TS when the overwhelming majority of funding opportunities and pathways to publication and promotion focus on TR (and also basic and/or clinical and/or population research) rather than TS. Given the centrality of education and career development within the CTSA program, and the current reality that of all NIH institutes and centers only NCATS is interested in both promoting and funding TS, we must ask ourselves as trainers: might we be setting them up for failure?

We were also interested to see that the proposals that were selected from our hub for the Element E project, the RC2 program, and the pilot program tended to be from senior investigators. Most applications from junior applicants were on the topics that were not deemed to be TS. Recognizing the risk of drawing generalizable inferences from our limited, single-institution data, this may suggest that senior scientists, because of their presumably broader research portfolio and experience, are better positioned to extend or reframe part of their existing work to be responsive to TS funding opportunities compared to their junior colleagues. Junior investigators may require mentoring, support, and resources beyond the levels they are currently receiving to successfully build a career in TS.

## Strategies to Facilitate TS

Our early efforts in launching TS programs at Einstein-Montefiore exposed several challenges: many investigators across scientific disciplines struggle to grasp the distinction between TS and TR, and even when they do, require substantial guidance and support from CTSA leaders to develop TS projects that lead to generalizable solutions across disease areas. Individualized coaching can help investigators “fine-tune” their projects and provide an environment for them to grasp the concepts of TS and envision how their expertise can be applied in this field. Concerns have also been expressed by the investigators, and in particular the junior faculty, regarding the path that their research would take and the uncertainty with future funding if they were to shift their focus from a particular target/disease/organ to align with the “holistic” TS focus. In addition to enhancing awareness to TS through outreach and education of the scientific community, it is essential that the CTSA program devise ways to not only attract but also retain the participation of investigators in TS research. Clearly, a multipronged strategy is needed to address the emerging barriers to broadly catalyzing TS. Plans and ideas that are being considered to facilitate TS at our institution include:

1. Defining TS priorities. To maximize impact with limited TS resources at our small CTSA hub, we will need to prioritize which TR roadblocks to address in the future. An inclusive “Town Hall” style discussion involving both the research community and local stakeholders would enable identification of areas that warrant prioritization and pursuit based on “unique institutional strengths,” consistent with the objectives of the current CTSA Program Announcement [3].
2. More TS educational content. Create additional TS educational content for our ICTR website, for example, video explaining TS, descriptions of currently funded TS projects, examples contrasting TS versus TR, and FAQ page for internal TS funding opportunities. With each TS-RFP, offer a live, 1-hour webinar to field questions about TS and the grant application process. Make a new section on the CTSA hub homepage about TS resources and activities.
3. TS presentations to institutional committees. Engage institutional leadership to further catalyze and improve understanding about TS throughout the hub. Give TS presentations to Department Chairs’ Committee, Research Leadership Council, individual departments, and hospital leaders to increase institutional awareness and support of TS resources and initiatives and to identify common TS goals and potential synergies across different centers (e.g., Cancer Center and Center for AIDS Research).
4. Recruiting TS researchers. Proactively reach out to researchers in different disciplines to spark project ideas and motivate them to pursue TS opportunities. Encourage them to examine the methods/processes routinely used in their disease-focused translational research and develop innovations that would enhance the impact, speed, and cost of not only their own work but also studies of other conditions. Provide additional guidance and mentoring to junior investigators on TS proposals. Identify and facilitate interactions between researchers with complementary expertise to advance TS projects.
5. TS consultations. Require investigators to consult with ICTR leaders prior to responding to a TS-RFP to ensure that topics align with program goals and obtain guidance on identifying and accessing TS resources (e.g., community engagement, biostatistics, informatics, and clinical trials). Extend the duration between TS funding announcement and application due dates to at least 3 months to allow for sufficient time for pre-submission consultations.
6. TS work-in-progress (WIP) and conference presentations. Presentations by TS pilot and other grant awardees to the research community would further increase awareness about TS. Presenters can also receive constructive feedback from and foster TS collaborations with attendees from diverse research backgrounds.
7. TS seminars. Expand and rebrand our monthly institution-wide Clinical Research Methodology Series to include lectures about TS topics (e.g., novel trial designs, enhancing trust between researchers and the community, etc.).
8. TS symposium. Organize a TS symposium with presentations by TS researchers at our and other hubs to share TS activities and stimulate cross-hub collaborations.
9. Disseminating TS. The ICTR dissemination and implementation team can work with the institution’s Public Relations and Communications offices to broadly disseminate TS research findings with potential to change clinical practice and improve health of all populations. Examples and results of impactful TS studies would also be highlighted and disseminated at CTSA and other meetings.
10. Identifying additional TS funding opportunities. Explore TS funding opportunities outside of NCATS. One potential funding source is the Advanced Research Projects Agency for Health (ARPA-H), a new independent entity within NIH to support high-impact solutions to challenging health problems. ARPA-H released an announcement in March 2023 seeking proposals for research to improve health outcomes across patient populations, communities, diseases, and health conditions. We encourage CTSA leaders, at the hubs and at NCATS, to advocate that ARPA-H support TS investigations as this would be consistent with ARPA-H’s goals of advancing “*high-potential, high-impact biomedical and health research that cannot be readily accomplished through traditional research or commercial activity … [and] … developing entirely new ways to tackle the hardest challenges in health*” [9].

## Conclusion

When NCATS was established in 2011, the first priority was to clearly define and promote its mission. This new center at NIH undertook a deliberate process to define translation, translational research, and translational science and emphasized that “*while it studies the first (translation) as a process, and performs the second (translational research), what distinguishes the Center from any other organization …is its focus on the third—translational science—as a discipline*” [1]. Twelve years later, however, these terms remain poorly understood. Chat-GPT’s response (August 3, 2023 version) to the query “What is Translational Science?” is emblematic of the limited and conflicting online information about TS: “*Translational science, also known as translational research, is an interdisciplinary approach to scientific inquiry that aims to bridge the gap between basic scientific discoveries and their practical applications in the real world. It focuses on moving scientific knowledge from the laboratory setting to improvements in human health and well-being.”*

For TS to truly flourish as its own discipline, persistent confusion surrounding different terminology related to translation must be resolved through substantial and coordinated efforts at both the local CTSA hub and national levels. Additionally, there is an urgent need to prioritize the availability of more TS funding opportunities and well-defined career paths for researchers pursuing TS. The field of implementation science gained traction only when several NIH institutes were willing to fund individual investigator grants on this topic; similar cross-NIH support is needed for TS to grow. Given the lack of clarity about TS that surfaced during our review of internal TS proposals, funding agencies would also need to provide additional pre-review orientation and training to ensure external grant reviewers have a clear understanding of TS. Other bottlenecks in fostering TS are sure to emerge as we and other CTSA hubs are further along in implementing our TS strategic goals. The collective sharing of experiences and innovative approaches to overcoming TS challenges that arise at our hubs is critical if the field is to expand and gain wider recognition and acceptance by the scientific and broader communities.
